# A loop-mediated isothermal amplification assay for *Schistosoma mansoni* detection in *Biomphalaria* spp. from schistosomiasis-endemic areas in Minas Gerais, Brazil

**DOI:** 10.1186/s13071-021-04888-y

**Published:** 2021-08-06

**Authors:** Silvia Gonçalves Mesquita, Floria Gabriela dos Santos Neves, Ronaldo Guilherme Carvalho Scholte, Omar dos Santos Carvalho, Cristina Toscano Fonseca, Roberta Lima Caldeira

**Affiliations:** 1 Grupo de Pesquisa em Helmintologia e Malacologia Médica, Instituto René Rachou, Fundação Oswaldo Cruz, Belo Horizonte, Minas Gerais Brazil; 2 Grupo de Pesquisa em Biologia e Imunologia Parasitária, Instituto René Rachou, Fundação Oswaldo Cruz, Belo Horizonte, Minas Gerais Brazil

**Keywords:** Schistosomiasis, *Biomphalaria*, *Schistosoma mansoni*, LAMP, Molecular diagnostics

## Abstract

**Background:**

Schistosomiasis a neglected tropical disease  endemic in Brazil. It is caused by the trematode *Schistosoma mansoni*, which is transmitted by snails of the genus *Biomphalaria*. Among measures used to control and eliminate schistosomiasis, accurate mapping and monitoring of snail breeding sites are recommended. Despite the limitations of parasitological methods, they are still used to identify infected snails. Loop-mediated isothermal amplification (LAMP) is a sensitive, rapid, and cost-effective diagnostic method for the identification of infected snails. In the work reported here, we aimed to validate the use of LAMP for the detection of *S. mansoni* in snails of the genus *Biomphalaria*.

**Methods:**

Snails were collected in five municipalities of the Mucuri Valley and Jequitinhonha Valley regions in the state of Minas Gerais, Brazil. Snails were pooled according to collection site and then squeezed for the detection of *S. mansoni* and other trematode larvae. Pooled snails were subjected to pepsin digestion and DNA extraction. Molecular assays were performed for species-specific identification and characterization of the samples. A previously described LAMP assay was adapted, evaluated, and validated using laboratory and field samples.

**Results:**

Using the parasitological method described here, *S. mansoni* cercariae were detected in snails from two collection sites, and cercariae of the family Spirorchiidae were found in snails from one site. The snails were identified by polymerase chain reaction (PCR)–restriction fragment length polymorphism (RFLP). *Biomphalaria glabrata*, the main snail host of *S. mansoni* in Brazil, was detected in 72.2% of the collection sites. *Biomphalaria kuhniana*, which is resistant to *S. mansoni* infection, was found in the remaining sites. Multiplex, low stringency (LS), and conventional PCR allowed the detection of positive snails in four additional sites. Trematodes belonging to the families Strigeidae and Echinostomatidae were detected by multiplex PCR in two sites. The LAMP assay was effective in detecting the presence of *S. mansoni* infection in laboratory (7 days post-infection) and field samples with no cross-reactivity for other trematodes. When compared to LS and conventional PCR, LAMP showed 100% specificity, 85.7% sensitivity, and a κ index of 0.88.

**Conclusions:**

Our findings suggest that LAMP is a good alternative method for the detection and monitoring of transmission foci of *S. mansoni*, as it was three times as effective as the parasitological examination used here for the detection of infection, and is more directly applicable in the field than other molecular techniques.

**Graphical abstract:**

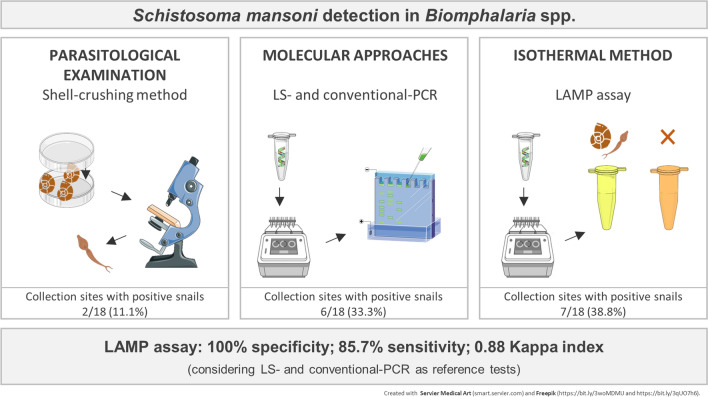

**Supplementary Information:**

The online version contains supplementary material available at 10.1186/s13071-021-04888-y.

## Background

Schistosomiasis is a parasitic disease that affects nearly 240 million people in the world. It is closely associated with poor sanitation and poverty, as these lead people to use contaminated water for domestic use and leisure [1]. It is estimated that over 25 million people live in areas of the Americas with a high risk of schistosomiasis. In Latin America, approximately 7.1 million people are infected with the etiological agent, *Schistosoma mansoni*, and 95% of them live in Brazil [2], where the northeastern and southeastern regions are the most affected [3]. *Biomphalaria* snails are essential for the maintenance of this parasite’s life cycle. Eleven species and one subspecies of *Biomphalaria* have been reported in Brazil, and three of them—*Biomphalaria glabrata*, *Biomphalaria tenagophila* and *Biomphalaria straminea*–have been found to be naturally infected with *S. mansoni* [4]. The presence of susceptible snail hosts in water bodies is crucial for the development of the parasite, and determines the distribution of schistosomiasis [5]. Knowledge regarding the geographic distribution of *Biomphalaria* snails in Brazil is being progressively updated, and demonstrates that these intermediate host species are spreading to new locations [3].

Among the control measures for the elimination of schistosomiasis, the surveillance of potential and active transmission foci, together with snail control measures, are highly recommended [6]. Traditionally, infected snail hosts are identified by either inducing cercarial shedding through artificial light exposure [7] or by using the shell-crushing method, followed by stereomicroscope examination to detect either cercariae or sporocysts in the snail tissue [8, 9]. However, many factors can limit the effectiveness of these parasitological methods. Inducing cercarial shedding is not applicable for the detection of early stages of snail infection, and can result in misidentification, as the larvae of other trematode species may be morphologically similar to each other, which means that an experienced person is required for their accurate identification. The shell-crushing method does not allow for the specific identification of sporocysts, and can damage the cercarial tissue, thus hindering the observation of differential morphological characters. In addition, neither method can be performed using dead snails [10–13].

In order to overcome these limitations, several alternative methods for the xenomonitoring of human schistosomes have been developed. Molecular approaches, such as conventional polymerase chain reaction (PCR) [14–17], low stringency-PCR (LS-PCR) [18], PCR–restriction fragment length polymorphism (PCR–RFLP) [19], nested PCR [17], multiplex PCR [20–22], real-time quantitative PCR [23, 24], DNA sequencing [25], and loop-mediated isothermal amplification (LAMP) [26–30] have all been shown to be more accurate and sensitive alternatives to the traditional microscope-based methods. Despite the high sensitivity and specificity of molecular methods, their cost and requirement for laboratory equipment have limited their usage for surveillance. In this context, the LAMP assay stands out as a promising method for the detection of *S. mansoni* infection in the field, as it does not require laboratory equipment such as PCR or electrophoresis apparatus [27].

The estimated annual cost of schistosomiasis in Brazil is over US $41 million, with more than 90% of this economic burden related to indirect costs (e.g. loss of productivity and wages due to sick leave, hospitalization, and premature death) [31]. This high economic burden and the persistence of schistosomiasis transmission in many areas in Brazil highlight the need for additional tools to control and eliminate this disease. In this study, the application of LAMP as described by Fernández-Soto et al. [32], with some adaptations, provided a rapid, accurate, specific, and sensitive isothermal method as an alternative approach for mapping and monitoring *S. mansoni* infection in *Biomphalaria* snail hosts.

## Methods

### Study area and malacological survey

The study was conducted in the municipalities of Franciscópolis (−17.9579, −42.0079) and Malacacheta (−17.84379959, −42.11119843) located in the Mucuri Valley (MV) region, and in the municipalities of Jequitinhonha (−16.4355, −41.0033), Joaíma (−16.653889, −41.030833) and Ponto dos Volantes (−16.752778, −41.503889) located in the Jequitinhonha Valley (JV) region. Both regions are endemic for schistosomiasis and are located in the state of Minas Gerais, Brazil (Additional file 1: Figure S1).

A total of 1001 snails were collected between July and August 2019 by members of the Helminthology and Medical Malacology Research Group (René Rachou Institute–Fiocruz Minas). All collection sites were georeferenced using global positioning system technology (Additional file 2: Table S1). The snails were transported to Fiocruz Minas, and some were deposited in the Fiocruz Collection of Medical Malacology (Fiocruz-CMM) after analysis.

### Parasitological examination and morphological identification of trematode larvae

The snails were separated into pools in the Lobato Paraense Mollusk Room (LPMR) at the René Rachou Institute–Fiocruz Minas according to their collection site (the number of snails in each pool is given in Additional file 2: Table S1) and then subjected to a shell-crushing/squeezing method for the detection of their natural infection with *S. mansoni* or other trematodes. The squeezed material was examined under a stereomicroscope to detect the presence of cercariae and/or sporocysts. The detected cercariae were isolated and part of the material was then observed under an optical microscope using non-permanent preparations for morphological identification. The morphological identification step was carried out according to the identification keys and descriptive works of different authors [33–36]. Cercariae were preserved in ethanol for future morphological and molecular analyses.

### Pepsin digestion and DNA extraction

The pooled squeezed snails were transferred to 50-ml centrifuge tubes labeled with the collection site code and subjected to pepsin digestion following the protocol of Wallace and Rosen [37] and sedimentation in accordance with the Baermann-Moraes method. The sediment was centrifuged for 20 min at 5000 *g*, the supernatant removed, and the remaining pellet cryopreserved at –80 °C until DNA extraction.

DNA extraction of the digested pooled snails was performed using the Wizard Genomic DNA Purification Kit (Promega, Madison, USA) in accordance with the manufacturer's instructions.

### PCR–RFLP for species-specific molecular identification of snails

Genomic DNA (gDNA) obtained from snail samples from all collection sites was used as the template for species-specific identification using a PCR–RFLP assay. The species-specific profiles generated after the digestion of the amplified internal transcribed spacer (ITS) fragment by the DdeI restriction enzyme (Promega) were used to identify the snails present in each pool; the profiles previously described by Caldeira et al. [19] were used as a reference. The results were visualized on silver-stained 6% polyacrylamide gels.

### Multiplex PCR for family-specific molecular identification of trematodes

In order to investigate the presence of trematode infection, the gDNA obtained from snail samples from all collection sites was used as the template for a trematode family-specific multiplex PCR in accordance with Mesquita et al. [38]. To compare the size of the amplified DNA fragments obtained from the field material, various positive controls were included using gDNA from cercariae belonging to the following trematode families: Clinostomidae, Echinostomatidae, Schistosomatidae, and Strigeidae. These samples were provided by the Laboratory of Trematode Biology, Department of Parasitology, Federal University of Minas Gerais, Brazil. Negative controls without DNA were included in each reaction. The resulting PCR products were visualized on silver-stained 6% polyacrylamide gels.

### LS-PCR for molecular detection of the presence of *S. mansoni* infection in snails

The gDNA obtained from snail samples from all the collection sites was used as the template for the LS-PCR for the detection of *S. mansoni* infection, which employed primers for the minisatellite region-mitochondrial DNA (mtDNA) protocol described by Jannotti-Passos et al. [18]. A sample from *S. mansoni* (10 ng/µl) was included as a positive control, and a negative control (no DNA) was also used. The amplification profile of the positive control was used as the standard for the amplification profile obtained from the unknown DNA samples.

### Conventional PCR for molecular detection of the presence of *S. mansoni* in snails

The outer primers F3 and B3 designed by Fernández-Soto et al. [32] were used in the conventional PCR to amplify a 203-bp mitochondrial fragment of the *S. mansoni* samples from MV and JV. Positive (10 ng/µl of *S. mansoni* gDNA) and negative controls (no DNA) were included. The reaction was carried out in a final volume of 25 µl containing 1X PCR Buffer (Invitrogen, USA), 1.5 mM MgCl_2_ (Invitrogen), 0.25 mM of each dNTP (Invitrogen), 2 pmol/µl of each primer (F3 and B3), 1.5 U of Platinum Taq DNA Polymerase (Invitrogen) and 2 µl of the DNA. The reaction was set up as follows: (i) initial denaturation at 94 °C for 1 min; (ii) 30 cycles of 20 s at 94 °C, 20 s at 60 °C and 30 s at 72 °C; (iii) final extension at 72 °C for 10 min. The PCR products were run and visualized on silver-stained 6% polyacrylamide gels.

### LAMP assay for specific detection of *S. mansoni* infection in snails

In order to detect *S. mansoni* infection, gDNA obtained from pooled snails was used as the template for the LAMP assay using the primers described by Fernández-Soto et al. [32] targeting the minisatellite region of the mtDNA, with adaptations of the established protocol. Briefly, the optimized reaction was carried out in a final volume of 25 µl as follows: 1X Isothermal Amplification Buffer [20 mM Tris–HCl at pH 8.8, 50 mM KCl, 10 mM (NH_4_)_2_SO_4_, 2 mM MgSO_4_, 0.1% Tween20; New England Biolabs, MA], 8 mM of supplementary MgSO_4_ (New England Biolabs), 1.4 mM of each dNTP (Invitrogen), 40 pmol/µl of each inner primer (FIP/BIP), 5 pmol/µl of each outer primer (F3/B3), 1 M betaine (Sigma, USA), 8 U of Bst 2.0 WarmStart DNA polymerase (New England Biolabs), and 2 µl of the DNA. The reaction tubes were incubated at 65 °C for 50 min, and then heated at 80 °C for 5 min to stop the reaction. The amplification products were detected using silver-stained 6% polyacrylamide gels. The results were also visualized by color change after the addition of 2 µl of SYBR Green I ×1000 (Life Technologies, CA) by the naked eye (positive, yellow-green; negative, orange) and by exposure to ultraviolet light (positive, fluorescent; negative, non-fluorescent).

In order to access the specificity of the modified assay, gDNA from trematodes commonly found parasitizing *Biomphalaria* snails in the Neotropical region was used as the template for the reaction described above. Cercariae samples of the following trematode families were used for this purpose: Clinostomidae, Echinostomatidae, Strigeidae, Spirorchiidae, Diplostomidae and Notocotylidae. These samples were provided by the Laboratory of Trematode Biology, Department of Parasitology, Federal University of Minas Gerais, Brazil. *B. glabrata* positive and negative for *S. mansoni* infection, as well as adult worms of *S. mansoni*, were also used (samples provided by Fiocruz-CMM).

The analytical detection limit of the LAMP assay was determined by serial dilutions of *S. mansoni* from 10 ng/µl to 0.1 fg/µl.

### Validation of the LAMP assay using laboratory and field samples

The validation step using laboratory samples evaluated the capacity of the assay to detect different stages of infection including in pools with different proportions of negative and positive snails. For this, *B. glabrata* snails were obtained from the LPMR, and the following squeezed samples were prepared: single snails obtained at either 1, 7, 14 or 28 days post-infection; a pool containing 20 negative snails and one snail at the pre-patent period of infection; and another pool containing 20 negative snails and one snail that was shedding cercariae. The extracted gDNA was used as the template for the optimized LAMP assay.

For validation using field samples, the gDNA from snails collected at the MV and JV regions was used as the template for the LAMP assay.

### Statistical analysis

To analyze the agreement between diagnostic tests, the κ index and its 95% confidence interval were calculated using the GraphPad online tool (www.graphpad.com/quickcalcs/kappa1/). The Landis and Koch [39] scale of agreement was used to analyze the data.

The sensitivity and specificity of the LAMP assay were calculated using a combination of the results from the LS-PCR and conventional PCR as references and the following formulas: sensitivity = (number of LAMP-positive results/number of infected snails) × 100; specificity = (number of LAMP-negative results/number of non-infected snails) × 100.

## Results

### Parasitological examination and morphological identification of trematode larvae

**Fig. 1:**
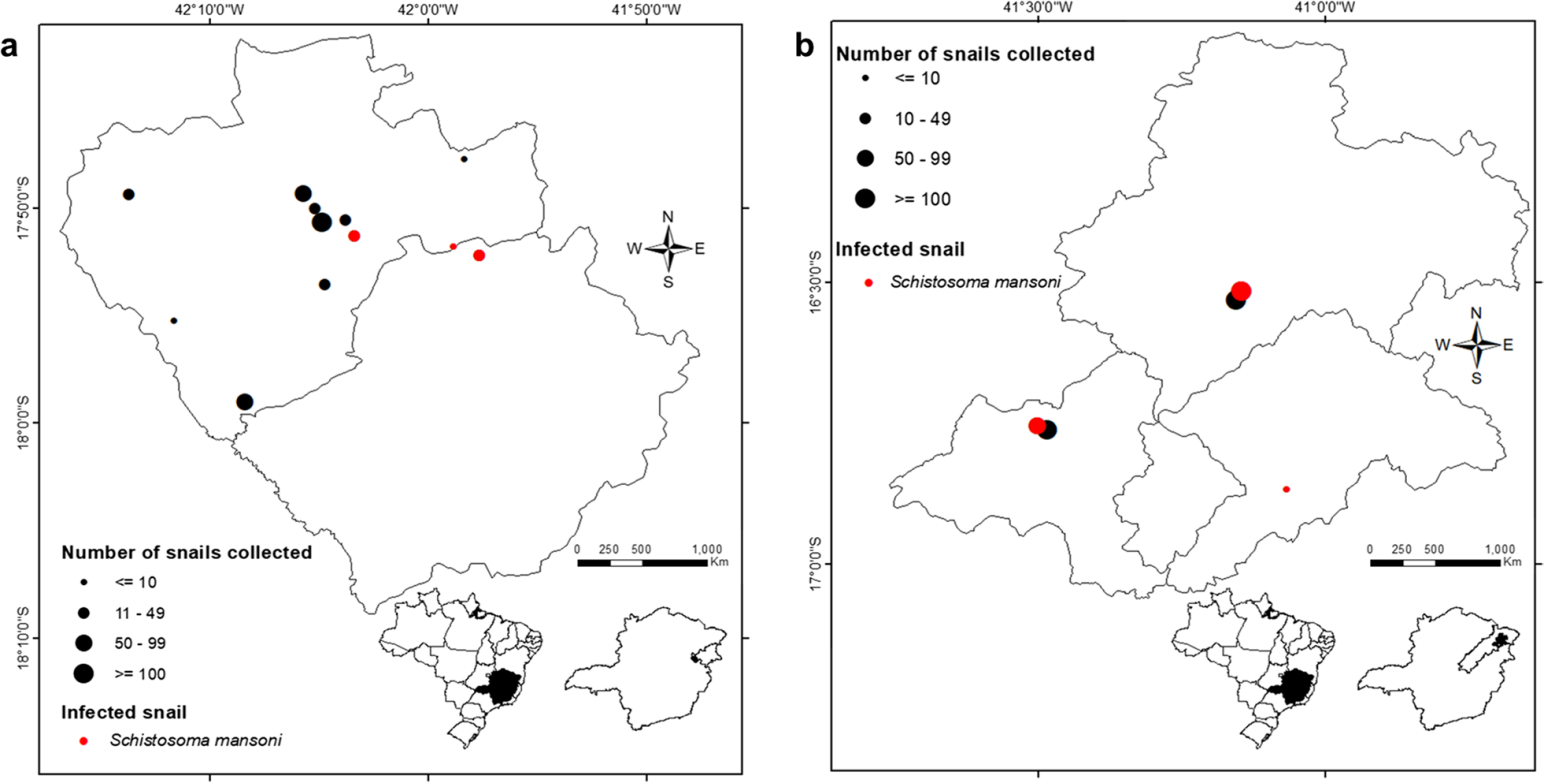
Maps of the study areas. The* dots* represent the collection sites and the* size of the dots* the population density of the snails in **a** the Mucuri Valley (MV) region and **b** the Jequitinhonha Valley (JV) region.* Red dots* represent the locations for which *Schistosoma mansoni* infection was detected in snails by molecular methods

A total of 399 snails were collected from 13 collection sites in MV. In the JV, 602 snails were collected from five collection sites (Fig. 1). Observation under a stereomicroscope after the shell-crushing examination demonstrated the presence of *S. mansoni* cercariae in snails from collection sites MV41 and JV04. The presence of cercariae belonging to the Spirorchiidae was observed in snails from collection site JV03.

### Species-specific molecular identification of the snails

The *B. glabrata* ITS-DdeI restriction profile was observed in snails from collection sites MV03, MV16, MV40, MV41, MV45, MV49, MV52, and MV65, and from all collection sites in JV. *Biomphalaria kuhniana* ITS-DdeI restriction profile was observed in snails from collection sites MV07, MV20, MV34, MV37, and MV39.

### Molecular investigation of the presence of four different families of trematodes in *Biomphalaria* snails

**Fig. 2:**
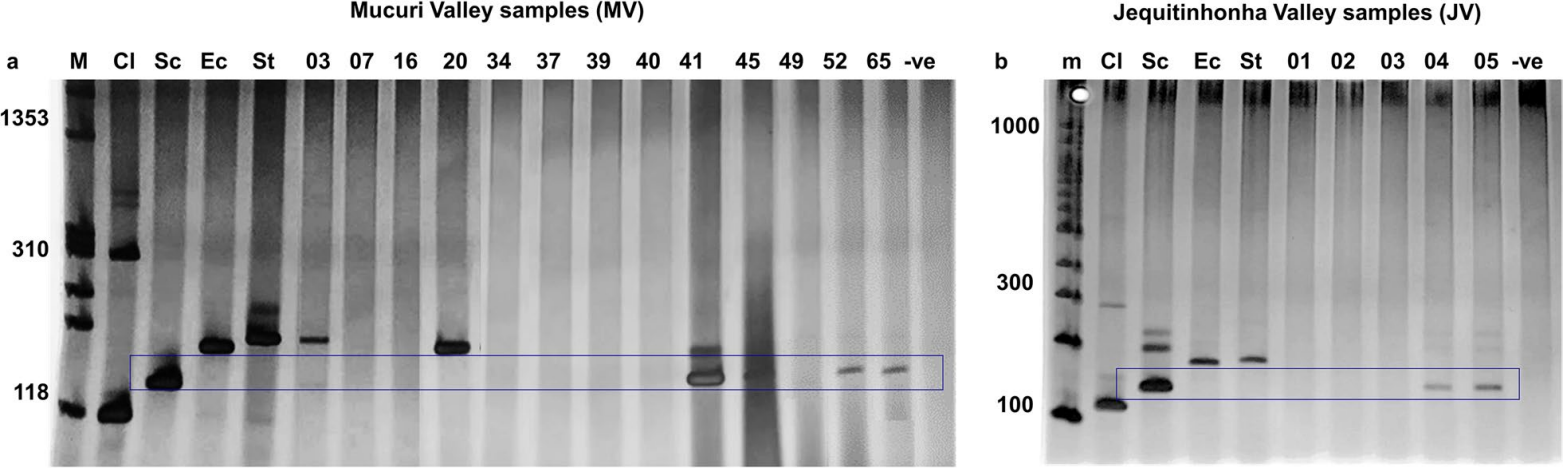
Multiplex polymerase chain reaction (PCR) results. Investigation of the presence of trematode infection in snails collected in **a** MV and **b** JV. Family Schistosomatidae DNA was detected in snails from collection sites MV41, MV45, MV52, MV65, JV04 and JV05. Co-infection with members of families Strigeidae and Schistosomatidae was detected for collection site MV03. Family Echinostomatidae DNA was detected for collection site MV20. *M* PhiX174 HaeIII marker,* Cl* cercariae belonging to the family Clinostomidae,* Sc* cercariae belonging to the Schistosomatidae,* Ec* cercariae belonging to the Echinostomatidae,* St* cercariae belonging to the Strigeidae,* −ve* negative control, *m* 100-bp marker; for other abbreviations, see Fig. 1

A multiplex PCR was conducted to detect the presence of infection by parasites of the Clinostomidae, Echinostomatidae, Schistosomatidae, and Strigeidae in the field-collected snails. Using the gDNA extracted from the pooled snails, it was possible to detect the presence of cercariae belonging to the Strigeidae and Schistosomatidae in *B. glabrata* from collection site MV03, the Echinostomatidae in *B. kuhniana* from collection site MV20, and the Schistosomatidae in *B. glabrata* from collection sites MV41, MV45, MV52, MV65, JV04, and JV05 (Fig. 2a, b).

### Molecular detection of *S. mansoni* in snails using LS-PCR and conventional PCR

**Fig. 3:**
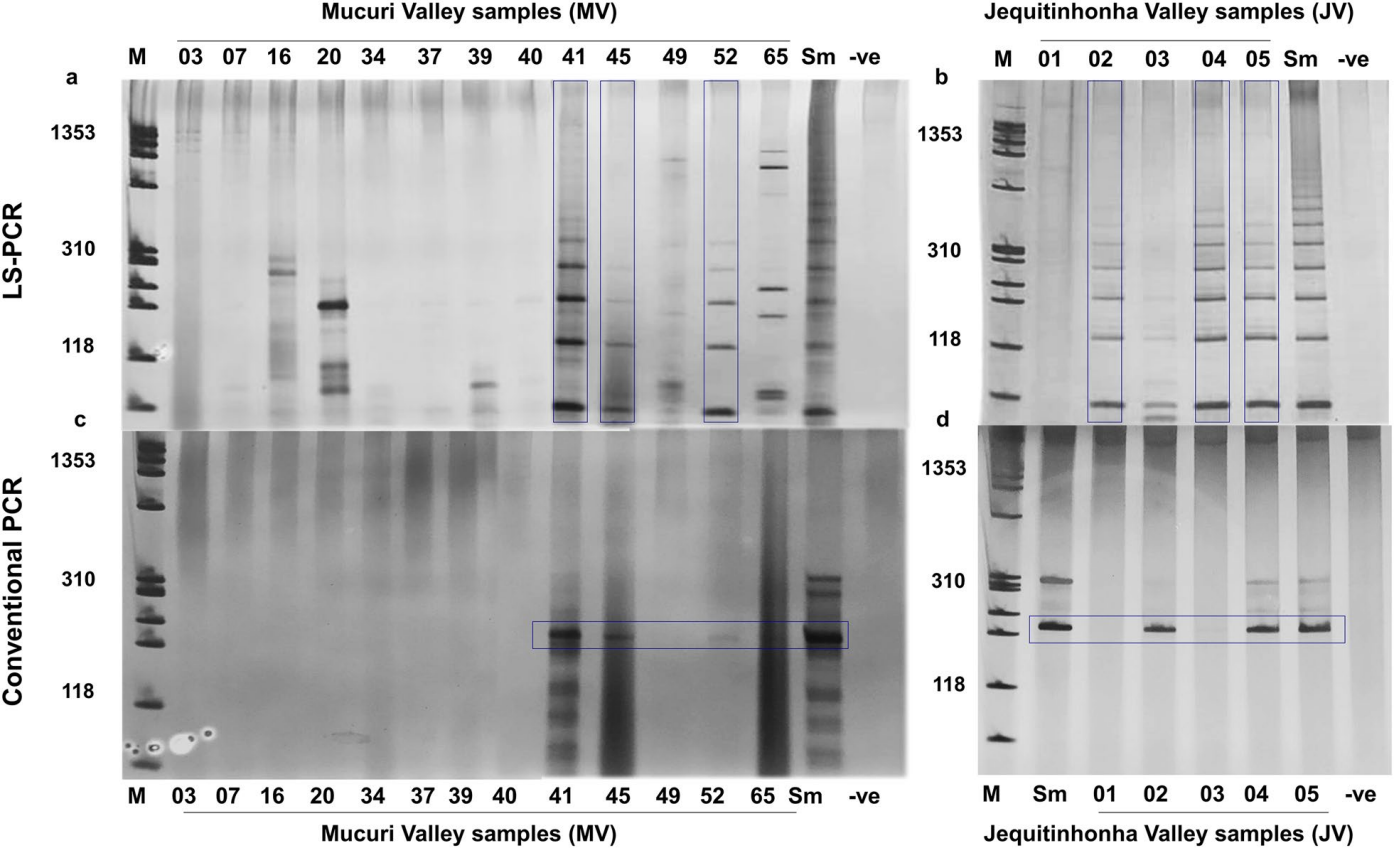
Detection by low stringency (LS)-PCR of *S. mansoni* infection in snail samples from **a** MV and **b** JV, and by conventional PCR using snail samples from **c** MV and **d** JV. Infection was found for the collection sites MV41, MV45, MV52, JV02, JV04, and JV05 by both methods.* Sm* *S. mansoni, B+* *B. glabrata* infected with *S. mansoni*,* B-* *B. glabrata* not infected with *S. mansoni*; for other abbreviations, see Figs. 1 and  2

The amplification pattern for *S. mansoni* generated after LS-PCR was observed for snails from collection sites MV41, MV45, and MV52, and JV02, JV04, and JV05 (Fig. 3a, b). The conventional PCR amplification of a mitochondrial fragment using the outer primers described by Fernández-Soto et al. [32] under the conditions standardized in the current study (Additional file 3: Figure S2) gave the same results as the LS-PCR. *S. mansoni* infection was detected in *B. glabrata* from collection sites MV41, MV45, and MV52MV, and JV02, JV04, and JV05 (Fig. 3c, d).

### Use of a LAMP assay to detect *S. mansoni* in *Biomphalaria* snails

**Fig. 4:**
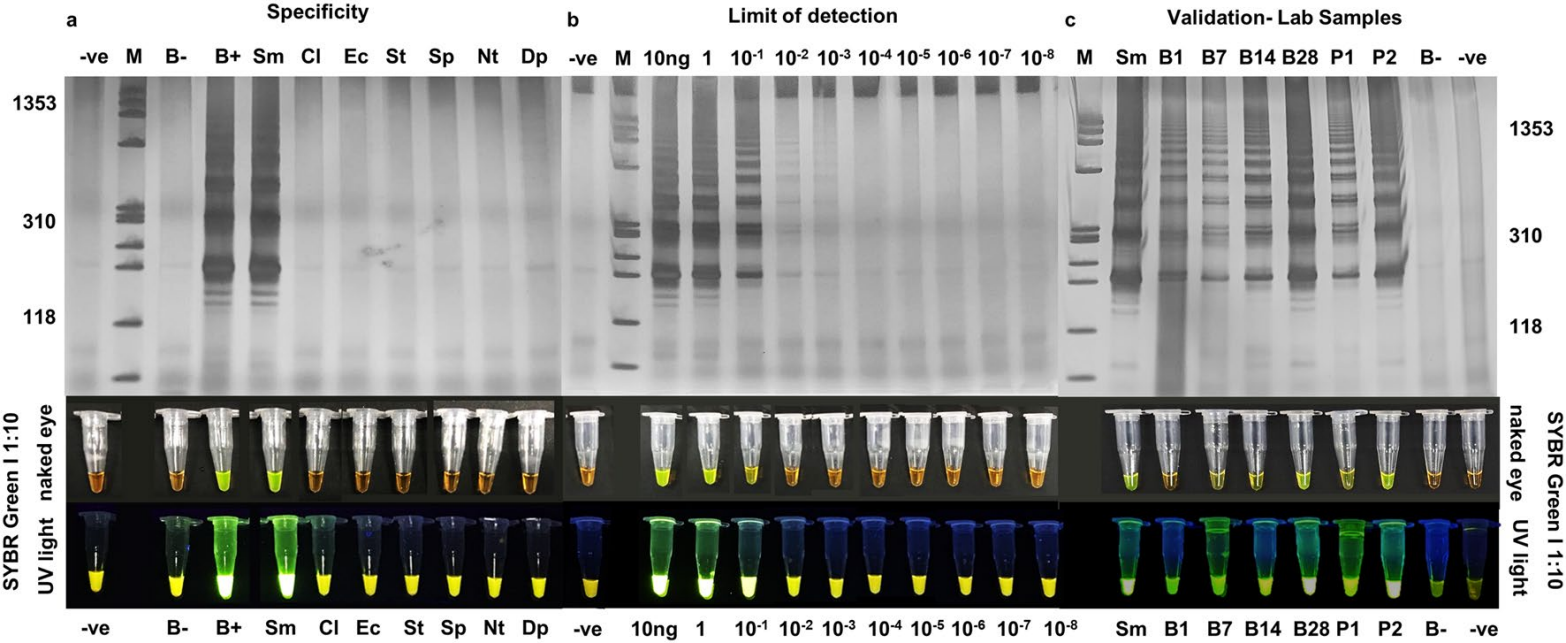
Optimization and validation of the loop-mediated isothermal amplification (LAMP) assay. LAMP products were visualized either by silver-stained 6% polyacrylamide gels or visual inspection of reaction tubes, with a 1:10 dilution of SYBR Green I, by the naked eye (positive, yellow-green; negative, orange) or exposure to ultraviolet (*UV*) light (positive, fluorescent; negative, non-fluorescent). **a** Specificity analysis of the optimized LAMP assay. No cross-reactivity was detected. **b** Analytical limit of detection. Optimized LAMP detected up to 10^–1^ ng of *S. mansoni* DNA. **c** Validation of the optimized LAMP assay using snails maintained in the laboratory at different stages of infection and pooled conditions. *S. mansoni* DNA was detected in all conditions.* B−* Negative snail,* B+* *Biomphalaria* snail infected with *S. mansoni*,* Nt* cercariae belonging to the family Notocotylidae,* Dp* cercariae belonging to the family Diplostomidae,* B1* snail 1 day post-infection (dpi),* B7* snail 7 dpi,* B14* snail 14 dpi,* B28* snail 28 dpi,* P1* pool of 19 negative snails plus one snail at the pre-patent period of infection,* P2* pool of 19 negative snails plus one snail shedding cercariae; for other abbreviations, see Figs.  2 and 3

We found that with some modifications to the Fernández-Soto et al. [32] protocol, the LAMP assay was effective in detecting *S. mansoni* infection in snails, with no cross-reaction with other trematode species that also parasitize *Biomphalaria* spp. (e.g. Clinostomidae, Diplostomidae, Echinostomatidae, Notocotylidae, Spirorchiidae and Strigeidae) (Fig. 4a). The assay had a detection limit of 0.1 ng of the parasite DNA (Fig. 4b). The assay was also able to detect infection by *S. mansoni* in laboratory samples when polyacrylamide gels or SYBR Green I were used to visualize the amplification products. When visual inspection employing SYBR Green I (1000 X) was used, the infection could be detected as early as 7 days after exposure of the snail to the parasite (Fig. 4c).

### Applicability of the LAMP assay to field-collected *Biomphalaria* snails

**Fig. 5:**
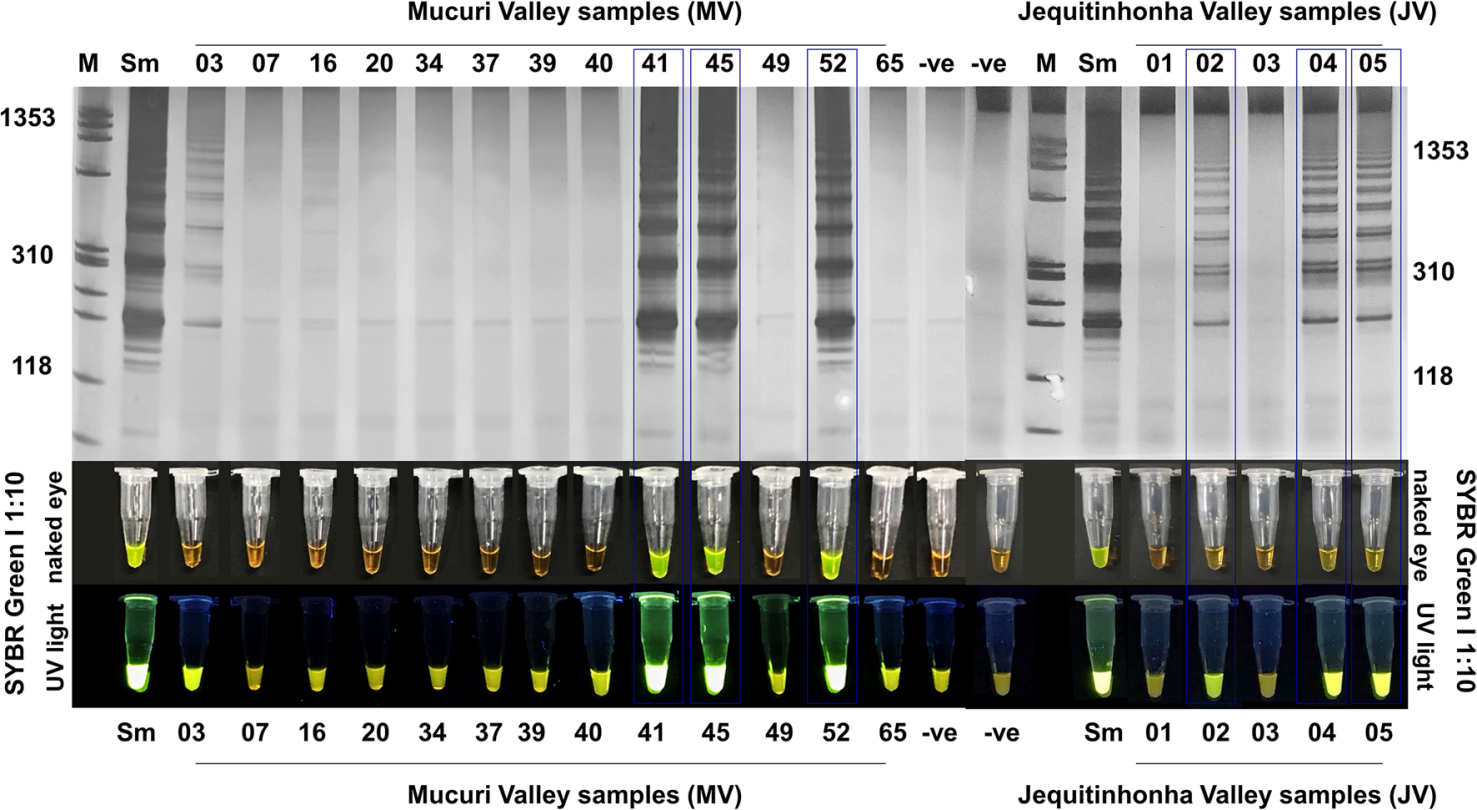
MV and JV snails examined by LAMP assay to detect *S. mansoni* infection. LAMP products were either visualized by using silver-stained 6% polyacrylamide gels or observed by visual inspection with the naked eye of reaction tubes with a 1:10 dilution of SYBR Green I (positive, yellow-green; negative, orange) or with UV light exposure (positive, fluorescent; negative, non-fluorescent). For abbreviations, see Figs. 1, 2, 3 and  4

Snails collected in the MV and JV were examined using LAMP. The LAMP amplification product was detected in *B. glabrata* from collection sites MV03, MV41, MV45, MV52, JV02, JV04, and JV05 when visualized using silver-stained 6% polyacrylamide gels. After the addition of 2 µl of SYBR Green I ×1,000, a color change was detected in snails from the same collection sites, except MV03 (Fig. 5) (Additional file 4: Table S2).

Considering LS-PCR and conventional PCR as reference tests, the optimized LAMP assay presented a sensitivity of 85.7% and specificity of 100%. The κ index showed an “almost perfect” agreement of 0.88 between the LAMP assay and the other molecular methods evaluated in this study. Therefore, our results showed that either LS-PCR, conventional PCR, and/or the LAMP assay could be used for xenomonitoring of transmission areas (Table 1), and that *S. mansoni* infection in *B. glabrata* from collection sites MV41, MV45, MV52, JV02, JV04, and JV05 could be detected by all three methods (Fig. 1).

**Table 1:** Comparison between the results of the low stringency-polymerase chain reaction (LS-PCR) together with the conventional PCR assay (*LS-PCR + conventional PCR*) and the loop-mediated isothermal amplification (*LAMP*) assay (κ index: 0.88)

		LAMP assay		
		Positive	Negative	Total
LS-PCR + conventional PCR	Positive	6	0	6
	Negative	1	11	12
	Total	7	11	18

## Discussion

In the present study, we identified active foci of *S. mansoni* transmission in six collection sites in the municipalities of Franciscópolis, Jequitinhonha, Joaíma, Malacacheta, and Ponto dos Volantes. Our results confirm that these areas, which are located in Minas Gerais, are endemic for *S. mansoni*. Around 70% of the areas endemic for *S. mansoni* in Brazil are located in Minas Gerais, and have been the subject of many studies over the years [3, 40–44]. Both MV and JV are very poor regions within Minas Gerais. Poverty contributes to the increased contact of individuals with contaminated water, as populations from underprivileged areas usually seek natural watercourses for domestic use and leisure activities, which leads to an increase in schistosomiasis transmission rates [41, 43]. Thus, monitoring snail breeding sites is vital for schistosomiasis control, but the methods commonly used to examine snails have some limitations and often give false negative results. Alternative methods, such as molecular approaches, enable the detection of infected snails at a higher level of accuracy than parasitological methods, which supports the need for additional molecular tools for the precise mapping and monitoring of areas endemic for *S. mansoni*.

In this study, a total of 1001 snails were collected from 18 sites in five municipalities of MV and JV in Minas Gerais, Brazil. Trematode larvae, detected by parasitological examination of the collected snails following shell-crushing, were found in 16.6% (3/18) of the collection sites, and most of them (two-thirds) were larvae of *S. mansoni*.

*B. glabrata* was identified in 72.2% (13/18) of the surveyed sites by PCR-RFLP. This result indicates that these are potential areas for the transmission of schistosomiasis, as this snail species is the main intermediate host of the parasite in Brazil due to its high compatibility with *S. mansoni* and wide distribution throughout the country [3, 45–47]. *B. kuhniana* snails were found in 27.8% (5/18) of the surveyed sites. Although this species is not thought to be important for the transmission of schistosomiasis, its close morphological similarity to the intermediate host *B. straminea* highlights the importance of correctly identifying these snail species in order to accurately map potential foci of schistosomiasis [48–50].

Some of the snails collected in this study were deposited in the Fiocruz-CMM, which was founded in 1993 and currently holds a collection of more than 16,800 snails from all over the world [51, 52]. According to the database of Fiocruz-CMM, which is available on the Centro de Referência em Informação Ambiental website [53], *Biomphalaria* snails have been previously reported in 14 of the 23 municipalities of MV. *B. glabrata*, *B. straminea*, and *Biomphalaria schrammi* have been collected in nine, 11, and two municipalities, respectively. The last survey in Fransciscópolis took place in 2013, and that in Malacacheta in 2015. In the present study, we have shown, to our knowledge for the first time, the presence of *B. kuhniana* in Malacacheta and *B. glabrata* in Franciscópolis. JV comprises 55 municipalities, and *Biomphalaria* snails have been reported in 33 of them. *Biomphalaria* *straminea* and *B. glabrata* have been previously collected in 21 and 18 municipalities respectively, while *B. kuhniana* and *B. tenagophila* in one municipality each. The most recent surveys were conducted in Jequitinhonha in 2006, in Joaíma in 2014, and in Ponto dos Volantes in 2012. The results for JV from our study match the data obtained from previous ones. We have found that the presence of *B. glabrata* has been maintained over the years in almost all of the areas in which we have conducted our surveys. Data from Fiocruz-CMM combined with our findings reinforce the importance of constant monitoring of these areas.

Trematode infection in snails was investigated using a multiplex PCR protocol that enables the differentiation of four important families commonly found parasitizing *Biomphalaria* snails [38, 54–56]. Schistosomatidae species were detected in 44.4% (8/18) of the study sites, while Echinostomatidae and Strigeidae were each found in 5.5% (1/18) of the sites. Snails from the collection sites MV41 and JV04 that were found shedding *S. mansoni* cercariae by parasitological examination had their infection confirmed by multiplex PCR through amplification of the 140-bp target corresponding to the Schistosomatidae. In five further sites in MV, and one in JV, Schistosomatidae infection in snails was also detected. Even though the amplification of Schistosomatidae DNA does not necessarily indicate the actual presence of *S. mansoni*, the results raise concern that these areas might be potential foci for schistosomiasis. As expected, no amplification was observed for the snails collected at site JV03, since the set of primers used does not cover members of the Spirorchiidae isolated from snails from this location. As the primers used in the multiplex PCR amplify DNA of four trematode families, it was not possible to confirm that snails from the remaining collection sites were not infected with other trematode families.

Both the LS-PCR and conventional PCR were able to detect the presence of *S. mansoni* in snails from 33.3% (6/18) of the surveyed sites. The LAMP assay used in this work revealed the presence of *S. mansoni* in snails from 38.8% (7/18) of the collection sites, when amplification was visualized using polyacrylamide gels, with an almost perfect κ index agreement with LS-PCR and conventional PCR, with 100% specificity, and 85.7% sensitivity. When reaction tubes were visually inspected after the addition of an intercalating dye, amplification was detected for snails from six collection sites, the same result as obtained by LS-PCR and conventional PCR. A very weak amplicon generated by the multiplex PCR corresponding to Schistosomatidae was detected in snails from collection site MV03, but no amplification was detected with LS-PCR and conventional PCR using this sample as the template. The apparent LAMP product of this sample, when visualized using a polyacrylamide gel, suggested the hypothesis that this technique was more sensitive than the LS-PCR and conventional PCR for the detection of *S. mansoni* infection in snails. LS-PCR can detect up to 1 pg of *S. mansoni* DNA [18], conventional PCR up to 0.01 pg (Additional file 3: Figure S2), while the LAMP assay had a limit of detection of 0.1 ng. The LAMP assay was expected to be less sensitive than the other evaluated methods, but the impact of PCR inhibitors usually present in field samples should be considered when evaluating the performance of each method. In addition, although several trematode samples were used to test the specificity of the optimized LAMP assay, samples of other species that belong to the Schistosomatidae were not used. The analysis of the results of the LAMP assay together with those of the multiplex PCR suggested cross-reactivity between members of the same family. In the Brazilian context, other schistosomes are not of great relevance to human health, as only *S. mansoni* causes schistosomiasis in Brazil. Avian schistosomes have been reported to cause cercarial dermatitis in the northern hemisphere, but this condition has not been reported thus far in Brazil [57–59]. As an alternative means of avoiding inconclusive results, we suggest that the visual inspection of reaction tubes by the naked eye should be prioritized, instead of running the LAMP products in gels. This strategy not only reduces the possibility of inconclusive results, but is also more applicable under field conditions when equipment is lacking.

The LAMP assay was first described in 2000 [60], and has since been used to detect many pathogens, including *S. mansoni*, but mostly in human samples [32, 61–65]. The applicability of LAMP for the screening of snails to characterize areas of *S. mansoni* transmission is very promising [66], and has been tested by several authors [26–30]. Molecular techniques can detect the presence of *S. mansoni* even when snails are not shedding cercariae, which should provide valuable information for surveillance services, as in many endemic areas collected snails rarely shed cercariae even though schistosomiasis transmission remains present. A failure to find cercarial shedding can be misleading, as it often gives the false impression of low or even absent transmission [66]. Although PCR-based methods can fulfill this gap in knowledge, these techniques are inappropriate for laboratories with limited resources, as they require expensive machinery and technical expertise, which increase the associated costs of each reaction. Among all the advantages associated with isothermal assays, the possibility of performing these tests directly in the field in laboratories with limited equipment is undeniable. We used the LAMP assay described by Fernández-Soto et al. [32]. When following the exact conditions described by Gandasegui et al. [29], non-specific amplification was detected for trematodes that belong to the Diplostomidae and Spirorchiidae (Additional file 5: Figure S3). These trematodes are very commonly found parasitizing *Biomphalaria* spp. in Brazil, and are morphologically similar to *S. mansoni*. Therefore, we considered relevant the maintenance of the specificity of the reaction, even though the adaptation of the original protocol resulted in a reduction in the analytical limit of detection (from 1 fg to 0.1 ng). Our findings confirmed that the amount of* S. mansoni* DNA that can be determined by the assay was sufficient for the presence of the parasite 7 days after exposure of the snails to eight miracidia, and in pooled samples, to be detected by visual inspection alone.

Using the LAMP assay, we determined a level of infection by *S. mansoni* in snails that was three times that determined by parasitological examination using a shell-crushing method, and revealed six active transmission areas for schistosomiasis, in MV and JV. Molecular methods also allowed the mapping of potential transmission foci through the identification of *B. glabrata* in much of the surveyed area, as demonstrated in the maps generated by this study.

## Conclusions

Parasitological methods based on the detection of *S. mansoni* larval forms in *Biomphalaria* snails are limited and are affected by the variation in disease prevalence in different regions, such that false negative results may often be obtained when these methods are applied. The LAMP assay, which performed as well as the other molecular approaches evaluated in this study, was a sensitive, specific, rapid, and precise diagnostic alternative to the latter. However, as an isothermal method, LAMP is relatively easy to perform directly in the field or in laboratories with limited equipment. Considering the challenges associated with the control of schistosomiasis, and even for the interruption of its transmission in endemic areas, mapping and monitoring transmission foci at a higher level of accuracy should help to improve decision-making processes to ensure more appropriate allocation of public funds and resources aimed at the elimination of schistosomiasis as a public health problem.

## Supplementary Information

**Additional file 1: Figure S1.** Images taken by Andrew Chamberlin during the snail survey in the Mucuri Valley (MV).

**Additional file 2: Table S1.** Description of all data for the surveyed sites in which snails were collected.

**Additional file 3: Figure S2.** The specificity and analytical limit of detection of the conventional polymerase chain reaction (PCR) standardized in this study.

**Additional file 4: Table S2.** Summary of the results obtained by using all of the methods employed in this work.

**Additional file 5: Figure S3.** Specificity analysis of the loop-mediated isothermal amplification (LAMP) assay following the original protocol.

## Data Availability

Not applicable.

## Abbreviations

LAMP: Loop-mediated isothermal amplification
PCR: Polymerase chain reaction
LS-PCR: Low stringency-polymerase chain reaction
PCR–RFLP: Polymerase chain reaction–restriction fragment length polymorphism
gDNA: Genomic DNA
ITS: Internal transcribed spacer
mtDNA: Mitochondrial DNA
Fiocruz: Oswaldo Cruz Foundation
CMM: Collection of Medical Malacology
LPMR: Lobato Paraense Mollusk Room
dpi: Days post-infection
MV: Mucuri Valley
JV: Jequitinhonha Valley

## References

[1] World Health Organization. Schistosomiasis (bilharzia). https://www.who.int/health-topics/schistosomiasis#tab=tab_1 Accessed 8 Nov 2020.

[2] Pan American Health Organization. Schistosomiasis. https://www.paho.org/en/topics/schistosomiasis Accessed 8 Nov 2020.

[3] Carvalho OS, Mendonça CLF, Marcelino JMR, Passos LKJ, Fernandez MA, Leal RS (2018). Distribuição geográfica dos hospedeiros intermediários do *Schistosoma mansoni* nos estados do Paraná, Minas Gerais, Bahia, Pernambuco e Rio Grande do Norte, 2012–2014. Epidemiol Serv Saúde..

[4] Paraense WL, Reis FA, Faria I, Katz N (1986). Distribuição dos Caramujos no Brasil. Modernos conhecimentos sobre esquistossomose mansônica.

[5] Barbosa CS (1992). Métodos de diagnóstico malacológico. Mem Inst Oswaldo Cruz.

[6] Allan F, Ame SM, Tian-Bi YNT, Hofkin BV, Webster BL, Diakité NR (2020). Snail-related contributions from the Schistosomiasis Consortium for Operational Research and Evaluation program including xenomonitoring, focal mollusciciding, biological control, and modeling. Am J Trop Med Hyg.

[7] Pellegrino J, Katz N (1968). Experimental chemotherapy of* Schistosomiasis mansoni*. Adv Parasitol.

[8] Paraense W, Correa L (1989). A potential vector of *Schistosoma mansoni* in Uruguay. Mem Inst Oswaldo Cruz.

[9] Gérard C, Balzan C, Théron A (1995). Spatial distribution patterns of the sporocyst infrapopulation of *Schistosoma mansoni* within its mollusc host (*Biomphalaria glabrata*): an unusual phenotype of aggregation. J Parasitol.

[10] Loker ES, Bayne CJ, Bukley PM, Kruse KT (1982). Ultrastructure of encapsulation of *Schistosoma mansoni* mother sporocysts by hemocytes of juveniles of the 10–R2 strain of *Biomphalaria glabrata*. J Parasitol.

[11] Schols R, Carolus H, Hammoud C, Mulero S, Mudavanhu A, Huyse T (2019). A rapid diagnostic multiplex PCR approach for xenomonitoring of human and animal schistosomiasis in a “One Health” context. Trans R Soc Trop Med Hyg.

[12] Born-Torrijos A, Poulin R, Raga JA, Holzer AS (2014). Estimating trematode prevalence in snail hosts using a single-step duplex PCR: how badly does cercarial shedding underestimate infection rates?. Parasites Vectors.

[13] Caldeira RL, Jannotti-Passos LK, Lira PM, Carvalho OS (2004). Diagnostic of *Biomphalaria* snails and *Schistosoma mansoni*: DNA obtained from traces of shell organic materials. Mem Inst Oswaldo Cruz.

[14] Hamburger J, Na H, Xu YX, Ramzy RM, Jourdane J, Ruppel A (1998). A polymerase chain reaction assay for detecting snails infected with bilharzia parasites (*Schistosoma mansoni*) from very early prepatency. Am J Trop Med Hyg..

[15] Farghaly A, Saleh AA, Mahdy S, Abd El-Khalik D, Abd El-Aal NF, Abdel-Rahman SA (2016). Molecular approach for detecting early prepatent *Schistosoma mansoni* infection in *Biomphalaria alexandrina* snail host. J Parasit Dis.

[16] Joof E, Andrus PS, Sowunmi K, Onyango VM, Wade CM (2020). Comparing PCR techniques against conventional cercarial shedding methods for detecting *Schistosoma mansoni* infection in *Biomphalaria* snails. Acta Trop..

[17] Gomes ALDV, Melo FL, Werkhauser RP, Abath FGC (2006). Development of a real time polymerase chain reaction for quantitation of *Schistosoma mansoni* DNA. Mem Inst Oswaldo Cruz.

[18] Jannotti-Passos LK, Vidigal THDA, Dias-Neto E, Pena SDJ, Simpson AJG, Dutra WO (1997). PCR amplification of the mitochondrial DNA minisatellite region to detect *Schistosoma mansoni* infection in *Biomphalaria glabrata* snails. J Parasitol.

[19] Caldeira RL, Teodoro TM, Jannotti-Passos LK, Lira-Moreira PM, Goveia CDO, Carvalho OS (2016). Characterization of South American snails of the genus *Biomphalaria* (Basommatophora: Planorbidae) and *Schistosoma mansoni* (Platyhelminthes: Trematoda) in molluscs by PCR-RFLP. Biomed Res Int.

[20] Jannotti-Passos LK, Magalhães KG, Carvalho OS, Vidigal THD (2006). Multiplex PCR for both identification of Brazilian *Biomphalaria* species (Gastropoda: Planorbidae) and diagnosis of infection by *Schistosoma mansoni* (Trematoda: Schistosomatidae). J Parasitol.

[21] Vidigal THDA, Magalhães KG, Kissinger JC, Caldeira RL, Simpson AJG, Carvalho OS (2002). A multiplex-PCR approach to identification of the Brazilian intermediate hosts of *Schistosoma mansoni*. Mem Inst Oswaldo Cruz.

[22] Pennance T, Archer J, Lugli EB, Rostron P, Llanwarne F, Ali SM (2020). Development of a molecular snail xenomonitoring assay to detect *Schistosoma haematobium* and *Schistosoma bovis* infections in their *Bulinus* snail hosts. Molecules..

[23] Zanardi VS, Barbosa LM, Simões FM, Thiengo SC, Blanton RE, Junior GR (2019). Prevalence of infection of *Biomphalaria glabrata* by *Schistosoma mansoni* and the risk of urban schistosomiasis mansoni in Salvador, Bahia, Brazil. Rev Soc Bras Med Trop..

[24] Fuss A, Mazigo HD, Mueller A (2020). Malacological survey to identify transmission sites for intestinal schistosomiasis on Ijinga Island, Mwanza, north-western Tanzania. Acta Trop..

[25] Bakuza JS, Gillespie R, Nkwengulila G, Adam A, Kilbride E, Mable BK (2017). Assessing *S. mansoni* prevalence in *Biomphalaria snails* in the Gombe ecosystem of western Tanzania: the importance of DNA sequence data for clarifying species identification. Parasites Vectors..

[26] Abbasi I, King CH, Muchiri EM, Hamburger J (2010). Detection of *Schistosoma mansoni* and *Schistosoma haematobium* DNA by loop-mediated isothermal amplification: identification of infected snails from early prepatency. Am J Trop Med Hyg.

[27] Hamburger J, Abbasi I, Kariuki C, Wanjala A, Mzungu E, Mungai P (2013). Evaluation of loop-mediated isothermal amplification suitable for molecular monitoring of schistosome-infected snails in field laboratories. Am J Trop Med Hyg.

[28] Gandasegui J, Fernández-Soto P, Hernández-Goenaga J, López-Abán J, Vicente B, Muro A (2016). Biompha-LAMP: a new rapid loop-mediated isothermal amplification assay for detecting *Schistosoma mansoni* in *Biomphalaria glabrata* snail host. PLoS Negl Trop Dis..

[29] Gandasegui J, Fernández-Soto P, Muro A, Barbosa SC, de Melo LF, Loyo R (2018). A field survey using LAMP assay for detection of *Schistosoma mansoni* in a low-transmission area of schistosomiasis in Umbuzeiro, Brazil: assessment in human and snail samples. PLoS Negl Trop Dis..

[30] Caldeira RL, Jannotti-Passos LK, Carvalhho ODS (2017). Use of molecular methods for the rapid mass detection of *Schistosoma mansoni* (Platyhelminthes: Trematoda) in *Biomphalaria* spp. (Gastropoda: Planorbidae). J Trop Med..

[31] Nascimento GL, Pegado HM, Domingues ALC, De Alencar Ximenes RA, Itria A, Cruz LN (2019). The cost of a disease targeted for elimination in Brazil: the case of schistosomiasis mansoni. Mem Inst Oswaldo Cruz..

[32] Fernández-Soto P, Gandasegui Arahuetes J, Sánchez Hernández A, López Abán J, Vicente Santiago B, Muro A (2014). A loop-mediated isothermal amplification (LAMP) assay for early detection of *Schistosoma mansoni* in stool samples: a diagnostic approach in a murine model. PLoS Negl Trop Dis..

[33] Pinto HA, Melo AL (2013). Larvas de trematódeos em moluscos do Brasil: panorama e perspectivas após um século de estudos. Rev Patol Trop.

[34] Ruiz JM (1952). Contribuição ao estudo das formas larvárias de trematódeos brasileiros. 2. Fauna de Santos, Estado de São Paulo. Mem Inst Butantan..

[35] Ruiz JM (1953). Contribuição ao estudo das formas larvárias de trematódeos brasileiros. 5. Descrição de três furcocercárias que ocorrem em planorbídeos hospedeiros do Schistosoma mansoni. Mem Inst Butantan..

[36] Schell SC. How to know the trematodes. W.C. Brown Co., editor. 1970.

[37] Wallace GD, Rosen L (1969). Techniques for recovering and identifying larvae of *Angiostrongylus cantonensis*. Malacology.

[38] Mesquita SG, Rodrigues-Luiz GF, Reis-Cunha JL, Cardoso MS, Mendonça CLF, Bueno LL (2020). A multiplex PCR protocol for rapid differential identification of four families of trematodes with medical and veterinary importance transmitted by *Biomphalaria* Preston, 1910 snails. Acta Trop..

[39] Landis JR, Koch GG (1977). The measurement of observer agreement for categorical data. Biometrics.

[40] Conceição MJ, Carlôto AE, de Melo EV, da Silva IM, Coura JR (2013). Prevalence and morbidity data on* Schistosoma mansoni* infection in two rural areas of Jequitinhonha and Rio Doce Valleys in Minas Gerais, Brazil. ISRN Parasitol..

[41] Sarvel AK, Oliveira ÁA, Silva AR, Lima ACL, Katz N (2011). Evaluation of a 25-year-program for the control of schistosomiasis mansoni in an endemic area in Brazil. PLoS Negl Trop Dis..

[42] Cabello RKSAA, Beck LCNH, Massara CL, Murta FLG, Guimarães RJPS, Pieri OS, et al. *Schistosoma mansoni* infection and related knowledge among schoolchildren in an endemic area of Minas Gerais, Brazil, prior to educational actions. Acta Trop. 2016;164:208–15. 10.1016/j.actatropica.2016.09.015 27647573

[43] Conceição MJ, Borges-Pereira J, Coura JR (2007). A thirty years follow-up study on schistosomiasis mansoni in a community of Minas Gerais. Brazil Mem Inst Oswaldo Cruz.

[44] Gazzinelli A, Velasquez-Melendez G, Crawford SB, LoVerde PT, Correa-Oliveira R, Kloss H (2006). Socioeconomic determinants of schistosomiasis in a poor rural area in Brazil. Acta Trop.

[45] Calasans TAS, Souza GTR, Melo CM, Madi RR, de Jeraldo LSV (2018). Socioenvironmental factors associated with *Schistosoma mansoni* infection and intermediate hosts in an urban area of northeastern Brazil. PLoS One..

[46] Paraense WL (2001). The schistosome vectors in the Americas. Mem Inst Oswaldo Cruz.

[47] Carvalho OS, Mendonça CLF, Teles HMS, Finau J, Caldeira RL, Scholte RGC, Mesquita SG. Moluscos hospedeiros intermediários de *Schistosoma mansoni* do Brasil. Belo Horizonte/Instituto René Rachou/Fiocruz. 2020.

[48] Dupuy V, Nicot A, Jarne P, David P (2009). Development of 10 microsatellite loci in the pulmonate snail *Biomphalaria kuhniana* (Mollusca, Gastropoda). Mol Ecol Resour.

[49] Caldeira RL, Jannotti-Passos LK, Carvalho OS (2009). Molecular epidemiology of Brazilian *Biomphalaria*: a review of the identification of species and the detection of infected snails. Acta Trop.

[50] Velásquez LE, Caldeira RL, Estrada V, Carvalho OS (2002). Morphological and polymerase chain reaction-restriction fragment length polymorphism characterization of *Biomphalaria kuhniana* and *Biomphalaria amazonica* from Colombia. Mem Inst Oswaldo Cruz.

[51] Aguiar-Silva C, Mendonça CLF, da Pinheiro CKPH, Mesquita SG, Carvalho OS, Caldeira RL (2014). Evaluation and updating of the Medical Malacology Collection (Fiocruz-CMM) using molecular taxonomy. SpringerPlus..

[52] Silva M, Chame M, Moratelli R (2018). Fiocruz biological collections: strengthening Brazil’s biodiversity knowledge and scientific applications opportunities. Biodivers Data J..

[53] Centro de Referência em Informação Ambiental. Fiocruz-CMM—Coleção de Malacologia Médica. http://splink.cria.org.br/manager/detail?resource=Fiocruz-CMM Accessed 25 Nov 2020.

[54] Fernández MV, Hamann MI, De Núñez MO (2016). New larval trematodes in *Biomphalaria* species (Planorbidae) from northeastern Argentina. Acta Parasitol.

[55] Alves Pinto H. Biologia e taxonomia de trematódeos transmitidos por moluscos dulceaquícolas na represa da Pampulha, Belo Horizonte, Minas Gerais, Brasil (Thesis). Belo Horizonte, MG: Universidade Federal de Minas Gerais;2013.

[56] Souza CP, Lima LC, Jannotti-Passos LK, Ferreira SS, Guimarães CT, Vieira IBF (1998). Limnic snails in the microregion of Belo Horizonte, Minas Gerais State, Brazil: a survey emphasizing on vectors of parasitosis. Rev Soc Bras Med Trop.

[57] Horák P, Kolářová L (2011). Snails, waterfowl and cercarial dermatitis. Freshwater Biol.

[58] Horák P, Mikeš L, Lichtenbergová L, Skála V, Soldánová M, Brant SV (2015). Avian schistosomes and outbreaks of cercarial dermatitis. Clin Microbiol Rev.

[59] Pinto HA, Mati VLT, Melo AL (2012). Dermatite cercariana por esquistossomatídeos de aves: É possível a ocorrência de casos no Brasil?. Rev Patol Trop.

[60] Notomi T, Okayama H, Masubuchi H, Yonekawa T, Watanabe K, Amino N (2000). Loop-mediated isothermal amplification of DNA. Nucleic Acids Res..

[61] Mwangi IN, Agola EL, Mugambi RM, Shiraho EA, Mkoji GM (2018). Development and evaluation of a loop-mediated isothermal amplification assay for diagnosis of *Schistosoma mansoni* infection in faecal samples. J Parasitol Res.

[62] García-Bernalt Diego J, Fernández-Soto P, Crego-Vicente B, Alonso-Castrillejo S, Febrer-Sendra B, Gómez-Sánchez A (2019). Progress in loop-mediated isothermal amplification assay for detection of *Schistosoma mansoni* DNA: towards a ready-to-use test. Sci Rep.

[63] Fernández-Soto P, Gandasegui J, Rodríguez CC, Pérez-Arellano JL, Crego-Vicente B, Diego JGB (2019). Detection of *Schistosoma mansoni*-derived DNA in human urine samples by loop-mediated isothermal amplification (LAMP). PLoS One..

[64] Lodh N, Mikita K, Bosompem KM, Anyan WK, Quartey JK, Otchere J (2017). Point of care diagnosis of multiple schistosome parasites: species-specific DNA detection in urine by loop-mediated isothermal amplification (LAMP). Acta Trop.

[65] Fernández-Soto P, Avendaño C, Sala-Vizcaíno A, Crego-Vicente B, Febrer-Sendra B, García-Bernalt Diego J (2020). Molecular markers for detecting *Schistosoma* species by loop-mediated isothermal amplification. Dis Markers..

[66] Hamburger J (2020). Molecular tools and schistosomiasis transmission elimination. Am J Trop Med Hyg.

