# Supplementary figures and images for: A loop-mediated isothermal amplification assay for Schistosoma mansoni detection in Biomphalaria spp. from schistosomiasis-endemic areas in Minas Gerais, Brazil
Source: Parasit Vectors. 2021 Aug 6;14:388. doi: 10.1186/s13071-021-04888-y (PMC8343921; doi:10.1186/s13071-021-04888-y)

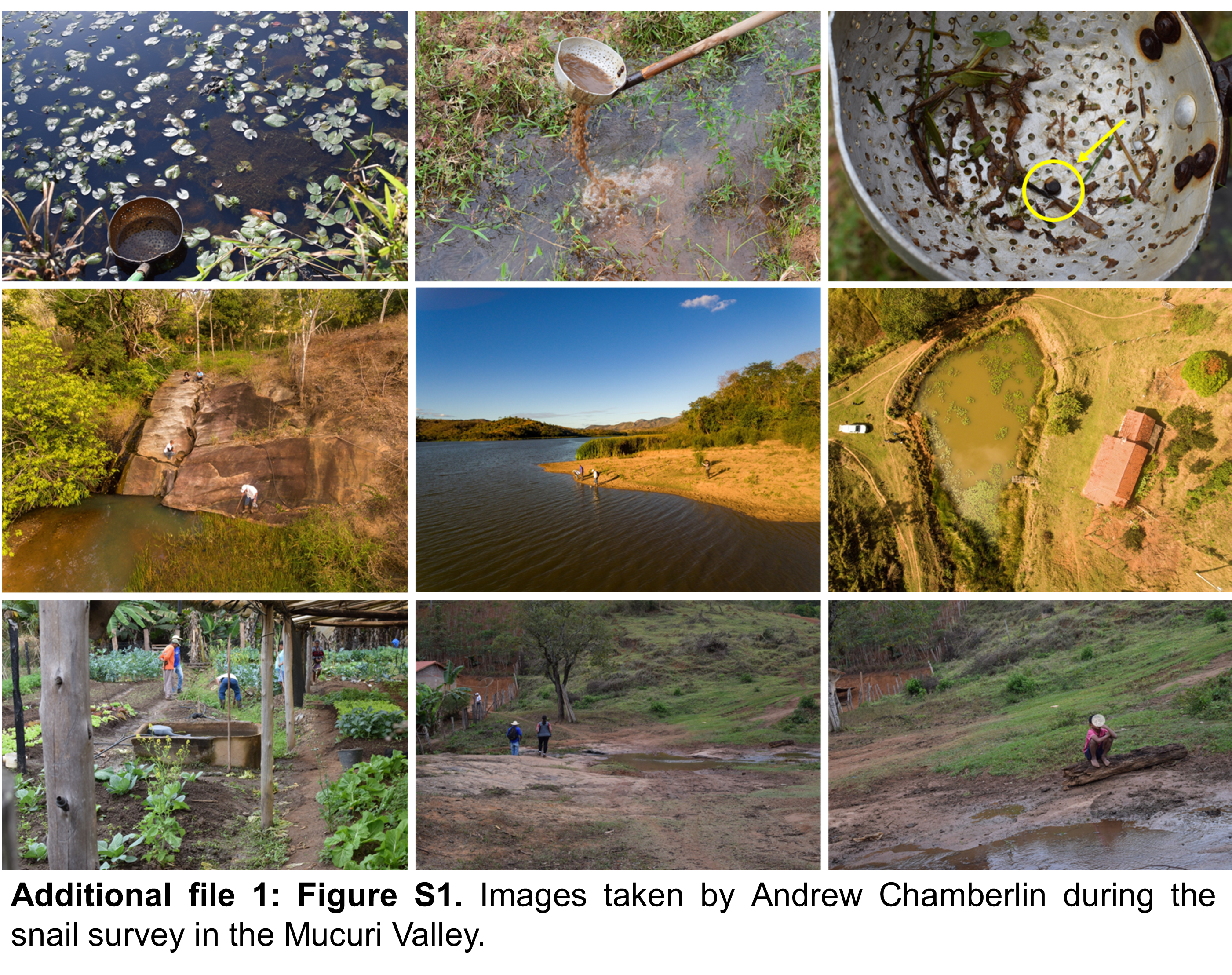

Supplement: Supplementary file 1 — Additional file 1: Figure S1. Images taken by Andrew Chamberlin during the snail survey in the Mucuri Valley (MV). [file 13071_2021_4888_MOESM1_ESM.tif]

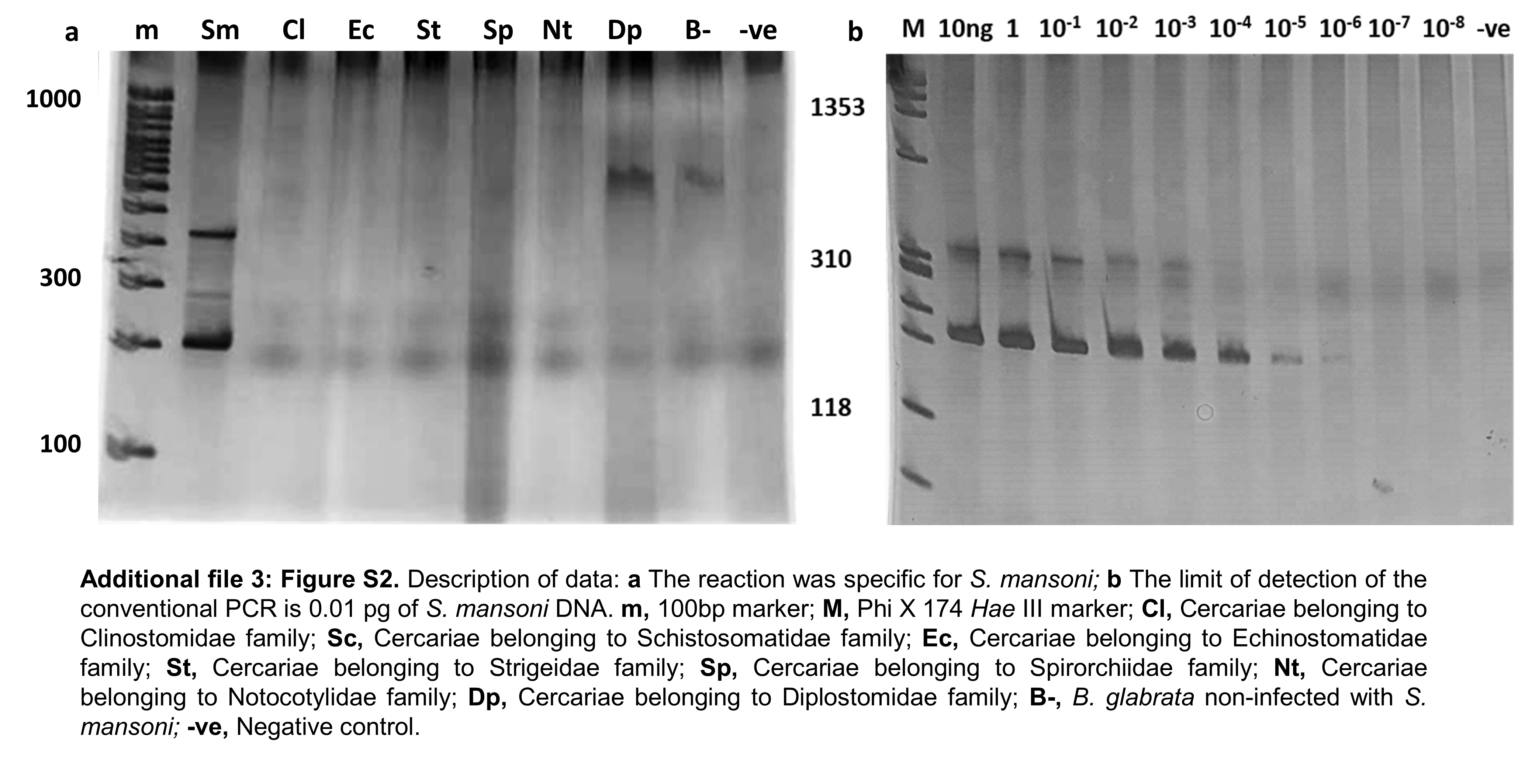

Supplement: Supplementary file 3 — Additional file 3: Figure S2. The specificity and analytical limit of detection of the conventional polymerase chain reaction (PCR) standardized in this study. [file 13071_2021_4888_MOESM3_ESM.tif]

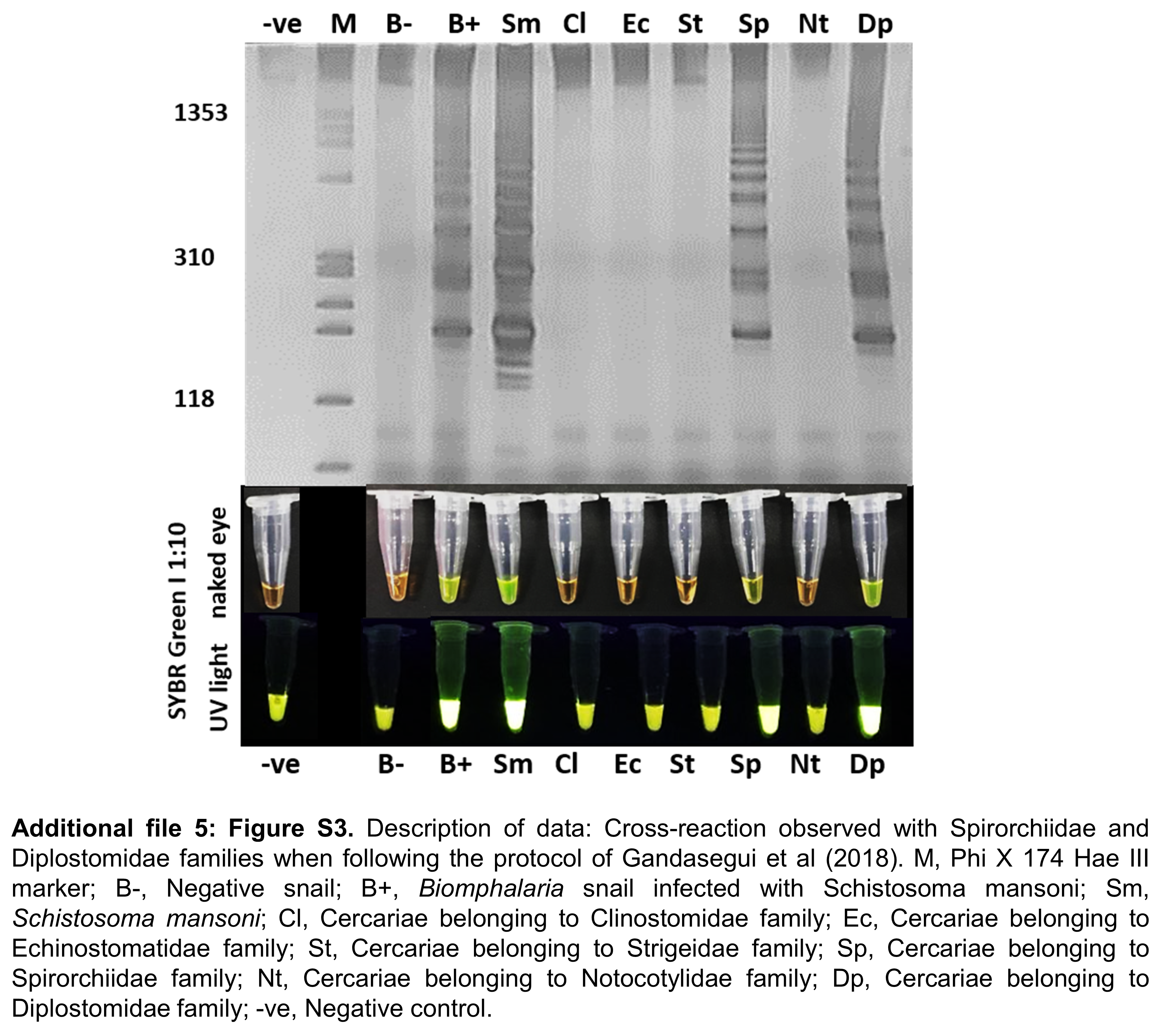

Supplement: Supplementary file 5 — Additional file 5: Figure S3. Specificity analysis of the loop-mediated isothermal amplification (LAMP) assay following the original protocol. [file 13071_2021_4888_MOESM5_ESM.tif]
