# Supplementary material for: A loop-mediated isothermal amplification assay for Schistosoma mansoni detection in Biomphalaria spp. from schistosomiasis-endemic areas in Minas Gerais, Brazil
Source: Parasit Vectors. 2021 Aug 6;14:388. doi: 10.1186/s13071-021-04888-y (PMC8343921; doi:10.1186/s13071-021-04888-y)
Supplement: Supplementary file 2 — Additional file 2: Table S1. Description of all data for the surveyed sites in which snails were collected. [file 13071_2021_4888_MOESM2_ESM.pdf]

Additional file 2: Table S1. Description of all data from the surveyed sites in which snails were collected.

Site Code	Collection Site	Municipality	Collection Date	Latitude	Longitude	Number of Snails Collected
**MV 03**	Santa Cruz Stream	Malacacheta	2019/07/22	-17.84179	-42.06258	33
**MV 07**	Serra Stream	Malacacheta	2019/07/22	-17.82212	-42.22862	21
**MV 16**	São José da Mata River	Malacacheta	2019/07/23	-17.82163	-42.09506	51
**MV 20**	Pond at the Monte Cristo allotment	Malacacheta	2019/07/23	-17.83321	-42.08592	20
**MV 34**	Pond at the Floresta Community	Malacacheta	2019/07/24	-17.89191	-42.07847	24
**MV 37**	São José da Mata River	Malacacheta	2019/07/24	-17.98381	-42.13998	50
**MV 39**	Índio Stream	Malacacheta	2019/07/24	-17.84418	-42.08073	12
**MV 40**	Índio Stream	Malacacheta	2019/07/25	-42.08068	-17.84399	151
**MV 41**	Spring at Lavra dos Rosas	Franciscópolis	2019/07/25	-17.86941	-41.96084	14
**MV 45**	Stream at Lavra dos Rosas	Malacacheta	2019/07/25	-17.86264	-41.98034	2
**MV 49**	Stream at Santo Antônio do Mucuri	Malacacheta	2019/07/25	-17.79498	-41.97259	5
**MV 52**	Santa Cruz Stream	Malacacheta	2019/07/26	-17.85454	-42.05589	15
**MV 65**	Trindade River	Malacacheta	2019/07/26	-17.92069	-42.19420	1
**JV 01**	Pilões River	Ponto dos Volantes	2019/08/13	-16.751015	-41.503429	118
**JV 02**	Pilões River	Ponto dos Volantes	2019/08/13	-16.754675	-41.502015	60
**JV 03**	Bom Jardim River	Jequitinhonha	2019/08/13	-16.520235	-41.15031	294
**JV 04**	Bom Jardim River	Jequitinhonha	2019/08/13	-16.51592	-41.14547	123
**JV 05**	Giru Stream	Joaíma	2019/08/13	-16.866549	-41.067264	07
**TOTAL**						**1001**
