# Supplementary material for: A loop-mediated isothermal amplification assay for Schistosoma mansoni detection in Biomphalaria spp. from schistosomiasis-endemic areas in Minas Gerais, Brazil
Source: Parasit Vectors. 2021 Aug 6;14:388. doi: 10.1186/s13071-021-04888-y (PMC8343921; doi:10.1186/s13071-021-04888-y)
Supplement: Supplementary file 4 — Additional file 4: Table S2. Summary of the results obtained by using all of the methods employed in this work. [file 13071_2021_4888_MOESM4_ESM.pdf]

Additional file 4: Table S2. Summary of the results obtained by all methods used in this work.

Site Code	Snail specie-specific identification	Parasitological exam	Trematode family-specific identification	Detection of the presence of *Schistosoma mansoni*		
	**PCR-RFLP**	**Morphological identification of the cercariae**	**PCR Multiplex**	**LS-PCR**	**PCR**	**LAMP**
**MV 03**	*B. glabrata*	-	Strigeidae and Schistosomatidae	-	-	Gel: Amplification<br>Dye: no color change
**MV 07**	*B. kuhniana*	-	-	-	-	-
**MV 16**	*B. glabrata*	-	-	-	-	-
**MV 20**	*B. kuhniana*	-	Echinostomatidae	-	-	-
**MV 34**	*B. kuhniana*	-	-	-	-	-
**MV 37**	*B. kuhniana*	-	-	-	-	-
**MV 39**	*B. kuhniana*	-	-	-	-	-
**MV 40**	*B. glabrata*	-	-	-	-	-
**MV 41**	*B. glabrata*	*S. mansoni* cercariae	Schistosomatidae	*S. mansoni*	*S. mansoni*	*S. mansoni*
**MV 45**	*B. glabrata*	-	Schistosomatidae	*S. mansoni*	*S. mansoni*	*S. mansoni*
**MV 49**	*B. glabrata*	-	-	-	-	-
**MV 52**	*B. glabrata*	-	Schistosomatidae	*S. mansoni*	*S. mansoni*	*S. mansoni*
**MV 65**	*B. glabrata*	-	Schistosomatidae	-	-	-
**JV 01**	*B. glabrata*	-	-	-	-	-
**JV 02**	*B. glabrata*	-	-	*S. mansoni*	*S. mansoni*	*S. mansoni*
**JV 03**	*B. glabrata*	Spirorchiidae cercariae	-	-	-	-
**JV 04**	*B. glabrata*	*S. mansoni* cercariae	Schistosomatidae	*S. mansoni*	*S. mansoni*	*S. mansoni*
**JV 05**	*B. glabrata*	-	Schistosomatidae	*S. mansoni*	*S. mansoni*	*S. mansoni*
